# Radiosensitising effect of electrochemotherapy with bleomycin in LPB sarcoma cells and tumors in mice

**DOI:** 10.1186/1471-2407-5-115

**Published:** 2005-09-16

**Authors:** Simona Kranjc, Maja Cemazar, Alenka Grosel, Marjeta Sentjurc, Gregor Sersa

**Affiliations:** 1 Institute of Oncology Ljubljana, Department of Experimental Oncology, Zaloska 2, SI-1000 Ljubljana, Slovenia; 2 Jozef Stefan Institute, Jamova 39, SI-1000 Ljubljana, Slovenia

## Abstract

**Background:**

Bleomycin is poorly permeant but potent cytotoxic and radiosensitizing drug. The aim of the study was to evaluate whether a physical drug delivery system – electroporation can increase radiosensitising effect of bleomycin *in vitro *and *in vivo*.

**Methods:**

LPB sarcoma cells and tumors were treated either with bleomycin, electroporation or ionizing radiation, and combination of these treatments. *In vitro*, response to different treatments was determined by colony forming assay, while *in vivo*, treatment effectiveness was determined by local tumor control (TCD_50_). Time dependence of partial oxygen pressure in LPB tumors after application of electric pulses was measured by electron paramagnetic oxyimetry.

**Results:**

Electroporation of cells *in vitro *increased radiosensitising effect of bleomycin for 1.5 times, *in vivo *radiation response of tumors was enhanced by 1.9 fold compared to response of tumors that were irradiated only. Neither treatment of tumors with bleomycin nor application of electric pulses only, affected radiation response of tumors. Application of electric pulses to the tumors induced profound but transient reduction of tumor oxygenation. Although tumor oxygenation after electroporation partially restored at the time of irradiation, it was still reduced at the level of radiobiologically relevant hypoxia.

**Conclusion:**

Our study shows that application of electric pulses to cells and tumors increases radiosensitising effect of bleomycin. Furthermore, our results demonstrate that the radiobiologically relevant hypoxia induced by electroporation of tumors did not counteract the pronounced radiosensitising effect of electrochemotherapy with bleomycin.

## Background

Bleomycin (BLM) is a glycopeptide antibiotic that is used in treatment of variety of human neoplasms, particularly lymphomas, squamous cell carcinoma and germ-cell tumors [1,2]. New understandings of mechanisms of its cytotoxicity have led to development of more potent and toxic therapeutic analogues [1]. Besides cytotoxic, BLM has also radiosensitising effect that was demonstrated in cells, experimental tumors as well as in the clinic [3-8].

Cytotoxicity of BLM once inside the cell is very high, but limited due to the poor uptake into the cells, which was shown to be receptor mediated endocytosis [9]. It has been shown that electroporation, a physical method of drug delivery into the cells and tissues, induces transient permeabilization of the cell membrane, thus enabling facilitated transport of BLM and other cytotoxic drugs like cisplatin into the cells, resulting in their increased cytotoxicity [10-12]. This combined treatment of chemotherapeutic drugs with hampered transport through the cell membrane and electroporation *in vivo *was termed electrochemotherapy (ECT). Its effectiveness in local tumor control was demonstrated in numerous animal tumor models and in several clinical studies in cancer patients with different tumors, malignant melanoma, sarcomas and carcinomas [13-15]. In addition, it was demonstrated that application of electric pulses to the tumor as well as normal tissues reduces blood flow and oxygenation [16-19].

The role of electroporation in radiosensitisation of tumors with cisplatin has already been shown. It was demonstrated that electroporation increased radiosensitising effect of cisplatin in two murine tumor models; EAT carcinoma and LPB sarcoma [20,21]. Feasibility of this approach was shown also in human adenocarcinoma skin metastases [22]. Furthermore, it was demonstrated that electroporation is effective in potentiating antitumor effectiveness of hypoxia-selective drug tirapazamine combined with tumor irradiation [23].

The aim of this study was to demonstrate the feasibility of this approach with another radiosensitising drug, BLM, which has proven its high effectiveness in ECT protocol. In addition, tumor oxygenation was measured after application of electric pulses to the tumors in order to determine the impact of reduced oxygenation in tumors on radiation response.

## Methods

### Cell line, tumors and animals

Murine sarcoma LPB cells, syngeneic to C57Bl/6 mice were used in the experiments. The cells were grown in Eagle minimum essential medium (EMEM) supplemented with 10% fetal calf serum (FCS) (Sigma, Chemical Co., St. Louis, MO, USA). Cells were routinely subcultured twice per week and maintained in a humidified atmosphere with 5% CO_2 _at 37°C. Inbred C57Bl/6 mice were purchased from the Institute of Pathology, University of Ljubljana (Slovenia). Mice were maintained at 21°C with natural day/night light cycle in a conventional animal colony. In the experiments mice of both sexes were used and distributed evenly between the groups. The average weight of mice subjected to treatment protocol was 22 g for female and 25 g for male mice.

Solid subcutaneous tumors were induced dorsolaterally by the injection of 1.3 × 10^6 ^viable tumor cells in EMEM supplemented with 2% FCS prepared from cell culture *in vitro*. The tumors reached approximately 40 mm^3 ^in volume in 10–12 days. Then, the mice were marked, divided randomly into experimental groups and subjected to specific experimental protocol. Treatment protocols were approved by the Ministry of Agriculture, Forestry and Food of the Republic of Slovenia No. 323-02-237/01, and are in compliance with the standards required by the UKCCCR guidelines.

### Drug

A stock solution (3 mg/ml) of BLM (Blenoxane, Bristol Myers Squibb Co., Princeton, NJ, USA) was prepared in phosphate buffered saline. Final concentration of BLM (0.28 μg/ml) was freshly prepared in EMEM before each experiment *in vitro*. *In vivo *experiments were performed using BLM at the dose of 0.5 mg/kg, which was daily prepared in 0.9% NaCl solution.

### Irradiation of cells and tumors

For irradiation an X-ray unit Darpac 2000 (Gulmay Medical Ltd, Shepperton, UK), operated at 220 kV, 10 mA, and with 0.55 mm Cu and 1.8 mm Al filtration was used. In experiments *in vitro*, cells (1 × 10^6 ^cells/ml EMEM) were irradiated in low attachment plates at a dose rate 2 Gy/min with graded doses (2–8 Gy) and thereafter plated in Petri dishes for colony forming assay. In experiments *in vivo*, tumors were irradiated at a dose rate 2.1 Gy/min with single graded doses (5–50 Gy). Mice were put into a holder for 6 mice on the X-ray unit with the apertures for the irradiation of the tumors; the rest of the body of the mice was protected by lead block, and by the lead holder for the mice. In the holders, mice were restrained, but not anaesthetized during irradiation. To ensure uniform dose distribution through the tumor volume, the tumors were exposed to irradiation by two opposing treatment fields through each of which 50% of the dose was delivered [21].

### Electrochemotherapy of cells and tumors

For electroporation, an electric pulse generator Jouan GHT 1287 (St. Herblain, France) delivering 8 square wave electric pulses of amplitude over distance ratio of 1200 V/cm, duration 100 μs at 1 Hz was used. In experiments *in vitro *90 μl of cell suspension (2.2 × 10^7 ^cells/ml) was mixed with 10 μl of BLM at a concentration of 0.28 μg/ml, as described previously [20]. Briefly, one half of the mixture was exposed to electric pulses and the other half served as a control for BLM treatment alone. Thereafter the cells were incubated for 5 min at room temperature in low attachment plates, diluted and plated on Petri dishes for colony forming assay. The survival of cells treated with ECT was normalized to electroporation treatment alone.

In *in vivo *experiments, ECT of tumors was performed as described previously [20]. Briefly, 3 min after intravenous injection of BLM (injection volume 150 μl), electric pulses were applied to the tumors using plate electrodes with 8 mm distance between them. Electrodes were placed on the skin overlying the tumor at the opposing margins and electric pulses delivered in two sets of 4 pulses in opposed directions by rotating the electrodes for 90°.

### Study design *in vitro*

**Figure 1:**
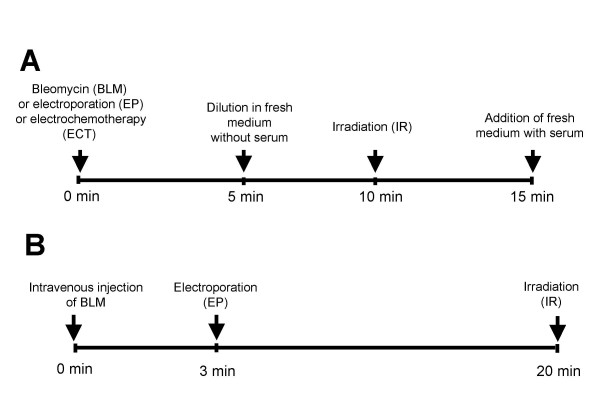
Schematic illustration of the experimental setting for the *in vitro *(A) and *in vivo *(B) study.

To determine whether electroporation increases radiosensitising effect of BLM *in vitro*, LPB cells were exposed to electric pulses in the presence of BLM and placed in low attachment plates for 5 min. Thereafter, 1 ml of EMEM was added and after additional 5 min cells were irradiated and plated in Petri dishes (Figure 1A). The survival of cells treated with different treatment combinations that included irradiation, were normalized to the appropriate control group, in order to demonstrate the interaction between the treatments on cell's survival. Survival of cells after electroporation and irradiation was normalized to the effect of electroporation, survival of cells after combination of BLM and irradiation was normalized to the effect of BLM, whereas when the cells were irradiated after ECT, cell survival data were normalized to the effect of ECT. All the data were pooled from 3 independent experiments performed in triplicates. The effect of treatments was assessed by comparison of IC_90 _values (drug concentration required to reduce cell survival for 90%). Enhancement factor (EF) was calculated on the basis IC_90 _values.

### Study design *in vivo*

To determine whether application of electric pulses increases radiosensitising effect of BLM, ECT was combined with local tumor irradiation with 20 min interval between the treatments (Figure 1B). Antitumor effectiveness of ECT combined with irradiation was evaluated by comparing the effects to single treatments or to the other treatment combinations: control untreated tumors, tumors treated with BLM, electroporation or irradiation only, tumors treated by ECT, and tumors treated with combination of BLM or electroporation and irradiation. Tumors were irradiated with single doses (5, 10, 15, 20, 25, 30, 35, 40, 45, 50 Gy). Each experimental group consisted of 3–7 mice and the data were pooled from 3 independent experiments.

### Assessment of tumor response

The tumor volume was determined by measuring three orthogonal tumor diameters (e_1_, e_2 _and e_3_) with Vernier calliper. Tumor volume was calculated by the formula V = π × e_1 _× e_2 _× e_3_/6. Tumor regression and regrowth was followed until the tumors grew up to 350 mm^3^, and then the animals were sacrificed. If the tumors regressed after therapy, animals were checked for the presence of the tumor in the irradiation field at 4–5 day intervals up to 100 days. The animals were considered cured if they were tumor free at day 100. Radiation dose needed to control 50% of irradiated tumors (TCD_50_) was used to determine response of tumors (included were 9–21 mice per irradiation dose). Dose response curves were computed by the logit method of analysis [24]. Enhancement factor (EF) was calculated based on TCD_50 _values.

### EPR oximetry measurements

EPR oximetry was used to measure partial oxygen pressure (pO_2_) in normal muscle, subcutaneous tissue, untreated LPB tumors as well as in the tumors subjected to electric pulses, as described previously [25]. Briefly, EPR oximetry is based on the fact that molecular oxygen is paramagnetic with two unpaired electrons and has a very rapid relaxation rate. It provides an effective relaxation mechanism to other paramagnetic species via Heisenberg spin exchange interaction. Consequently, the presence of oxygen influences both, spin-spin and spin-lattice relaxation times of the paramagnetic probe. Therefore, an increase in oxygen concentration increases the EPR spectra line-width, decreases the resolution of hyperfine structure, and decreases the microwave power at which the saturation of the EPR absorption lines occurs. All these parameters can be measured by EPR. Therefore, 3 small crystals of the oxygen sensitive paramagnetic probe lithium phtalocyanine (LiPc, 15 – 40 μm in diameter) were inserted into tumor (one in centre and one in periphery) and one in selected normal tissue (subcutaneous tissue or skeletal muscle) one day before the treatment. Immediately after the treatment, the EPR spectra were recorded continuously for 60 min and thereafter at each selected time point for 15 min. The mice were anaesthetized by intraperitoneal injection of a mixture of Domitor (1.0 mg/kg body weight; Pfizer GmbH, Karlsruhe, Germany) and 10% ketamine (75.0 mg/kg body weight; Veyx-Pharma GmbH, Schwarzenborn, Germany). During the anesthesia, warm air was used to keep the body temperature as close as possible to 37°C with variations of up to 0.5°C during single measurement. The measurements were performed on Varian E-9 EPR spectrometer, with a custom-made low frequency microwave bridge operating at 1.1 GHz and an extended loop resonator (11 mm in diameter), both designed by Professor T. Walczak (Darmouth Medical School, Hanover, NH). Typical spectrometer settings were: modulation frequency, 100 kHz; modulation amplitude not more than one-third of the peak-to-peak line-width, and scan range, 2 mT. The line width of the EPR spectra reflects the pO_2_, on the site of the paramagnetic probe and was determined from the calibration curve presented elsewhere, as the changes in the EPR spectrum can be calibrated with known concentration of oxygen [25].

### Statistical analysis

All data were tested for normality of distribution. The statistical differences between the treatment groups were assessed by a t-test after one-way ANOVA was performed and fulfilled. SigmaStat statistical software (SPSS inc.) was used for statistical analysis. P levels of less than 0.05 were taken as significant.

## Results

### Radiosensitisation of LPB cells *in vitro*

**Figure 2:**
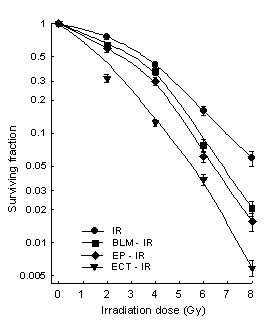
**Radiation survival curves of LPB cells **exposed to different irradiation doses only and to a combination with bleomycin (BLM; 0.28 μg/ml), electroporation and electrochemotherapy (ECT). Cell survival was determined using colony forming assay. Values are AM ± SEM (n = 9).

In order to determine the radiosensitising effect of BLM, LPB cells exposed to BLM alone or ECT were irradiated with graded X-ray doses up to 8 Gy (Figure 2). Exposure of cells either to BLM, electroporation or ECT statistically significantly increased radiation response of LPB cells. Increase in radiation response was the lowest when the cells were exposed to BLM (EF = 1.19), probably due to low BLM concentration and a very short exposure time. On the other hand, the radiosensitivity of LPB cells was greatly enhanced when the cells were pretreated with ECT (EF = 1.53). When cells were exposed to electric pulses prior to irradiation the enhancement factor was 1.25. Therefore, radiosensitising effect of ECT might not be due to just increased BLM delivery into the cells by electroporation, but also to radiosensitisation by electroporation of cells (Figure 2). Surviving fraction of LPB cells after pertinent control treatments BLM alone, electric pulses alone and ECT is presented in Table 1.

### Radiosensitisaton of LPB tumors *in vivo*

**Figure 3:**
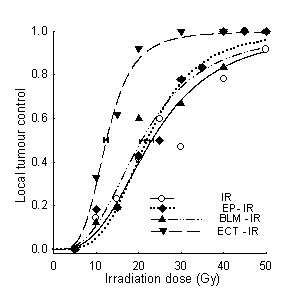
**Radiation dose response curves for local tumor control of LPB sarcoma tumors**. Tumors were treated with electrochemotherapy (ECT) 20 min prior to irradiation or in different combinations of irradiation either with bleomycin (BLM) given intravenously (0.5 mg/kg), or with application of 8 electric pulses to the tumors (electroporation – irradiation; EP-IR)). Groups consisted at least of 9 mice per irradiation dose.

Radiosensitising effect of ECT was evaluated also on subcutaneous LPB tumors in mice. As endpoints for evaluation of antitumor effectiveness were used tumor growth delay and local tumor control (TCD_50 _assay). Neither treatment of animals with BLM alone nor application of electric pulses to the tumors prior to irradiation of tumors had any effect on local tumor control, as observed by TCD_50 _values (Table 2, Figure 3). However, ECT of tumors statistically significantly increased radiation response of the tumors (Table 2, Figure 3). TCD_50 _value was reduced from 23.1 Gy in mice that were treated with irradiation only, to 12.4 Gy in mice that were treated with ECT prior to irradiation. Since the enhancement factor was 1.9, it is evident that electroporation of tumors significantly contributed to radiosensitisation of tumors with BLM, specifically because combined treatment of ECT with irradiation was statistically significantly more effective than treatment of tumors that were irradiated in combination with electroporation or BLM.

Antitumor effectiveness of pertinent control groups BLM alone, electric pulses alone and ECT is shown in Table 1. ECT of tumors had good antitumor effect and some of the tumors were cured (8.7%). Treatment with BLM only or application of electric pulses to the tumors had minimal effect on tumor growth and no tumor cures were obtained after either of the treatments.

Side effects associated with application of electric pulses were instantaneous contractions of muscles located beneath the site of treatment, which disappeared immediately after each pulse. Irradiation alone or combined with BLM, electric pulses and ECT resulted in hair loss, but none of the treatments induced skin desquamation.

### Tissue oxygenation

**Figure 4:**
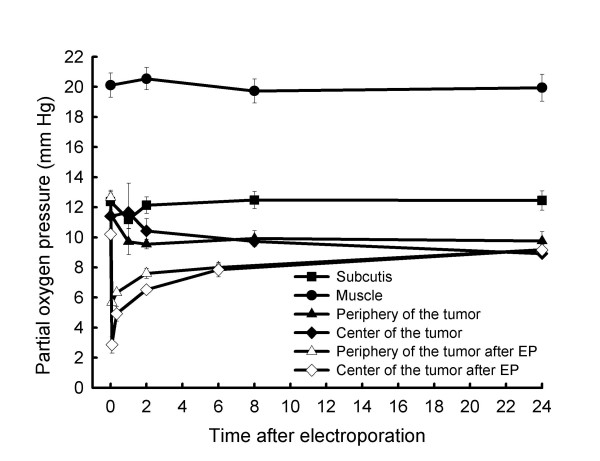
**Time dependence of partial oxygen pressure in normal tissues and LPB sarcoma tumors after application of electric pulses to the tumors**. pO_2 _measured in untreated LPB tumors at the same time points is also shown. Partial oxygen pressure was measured by EPR oximetry. Values are AM ± SEM (n = 7–10).

In order to determine whether electroporation of tumors can induce radiobiologically relevant hypoxia, that could reduce radiosensitising effect of ECT, pO_2 _was measured in the tumors after exposure to electric pulses. To determine whether reduction in tumor oxygenation is confined to the electroporated area (tumor), pO_2 _was measured in the same animal also in the normal tissues (skeletal muscle, subcutaneous tissue) that were not exposed to electric pulses. Application of electric pulses to the tumors statistically significantly reduced pO_2 _in the tumors (Figure 4). Five min after application of electric pulses to the tumors pO_2 _was lowered in the centre of the tumors down by 75% and in the periphery of the tumors down by 50%. Immediately thereafter, tumors started to reoxygenate; however 6 h after electroporation the oxygen level was still at 70% of pretreatment level and even after 24 h was not completely restored (Figure 4). Specifically, at 20 min after electroporation of the tumors, *i.e. *at the time of tumor irradiation, in the tumor centre as well as in the tumor periphery there was still reduced tumor oxygenation, at the level of radiobiologically relevant hypoxia (4.6 ± 0.6, 6.1 ± 0.3, respectively). In normal tissues; skeletal muscle and subcutaneous tissue pO_2 _was not affected by application of electric pulses to the tumors (Figure 4). In addition, pO_2 _was measured in untreated LPB tumors at the same time points to evaluate the whether tumor growth can affect tumor oxygenation. As shown on Figure 4, pO_2 _in untreated tumors did not change throughout the 24 h measurement.

## Discussion

This study shows that application of electric pulses to cells and tumors increases radiosensitising effect of BLM. As already demonstrated, electroporation of tumors increases BLM uptake into the tumor cells [10] and therefore this might be the principal mechanism of the increased radiosensitivity. Furthermore, our results demonstrate that the radiobiologically relevant hypoxia induced by electroporation of tumors did not counteract the pronounced radiosensitising effect of ECT.

Radiosensitising effect of BLM was demonstrated in many *in vitro *and *in vivo *studies, as well as in clinical trials [3,5-8,26,27]. Good potentiation of radiation response was found in some *in vitro *studies, even without use of drug delivery systems [4], which is in agreement with our results. Although with different concentration of BLM and incubation time used, the potentiation of radiation response was similar in the study of Leith *et al *and ours (enhancement factor 1.25 and 1.19, respectively). *In vivo *studies on mice demonstrated potentiation of radiation response with BLM when it was given in high doses up to 100 mg/kg [5,6]. At these doses radiation response was potentiated either after single irradiation dose or in fractionated regime up to 1.23-fold [5,6,8]. Our results showed that radiation response *in vivo *was not affected by BLM. The discrepancy in the response between our and other studies could be attributed to the much lower dose of BLM (0.5 mg/kg), which was used in our study.

Electroporation is one of the drug delivery systems which was shown to be effective in potentiation of BLM and cisplatin cytotoxicity [14]. *In vivo *ECT of tumors in experimental systems as well as in treatment of cancer patients is feasible and effective for local tumor control [13,15]. In this system low doses of drugs are needed, because electroporation of tumors *in vivo *increases drug accumulation in tumor cells from 2-fold for cisplatin and 4-fold for BLM [10,11]. Drug doses needed for effective local tumor control in ECT protocol are so low that they have minimal or no antitumor effectiveness without subsequent application of electric pulses to the tumors.

In order to reduce drug dosage for effective radiosensitisation of tumors several drug delivery systems can be used. Drug delivery systems, such as incorporation of the drug into liposomes or other vehicles and local drug administration were used to increase delivery of cisplatin to tumors [28-31], however to our knowledge there is no reports using drug delivery systems for *in vivo *radiosensitisation of tumors with BLM. Combination of ECT with cisplatin and irradiation has already been tested in cells, tumors in mice and a patient. The results demonstrated that ECT increased radiation response of cells and tumors, and that the predominant underlying mechanism was increased cisplatin accumulation in the cells of tumors [20-22]. The potentiation of radiation response by ECT with cisplatin was 1.4 compared to tumors that were treated with combined cisplatin and irradiation treatment, and 1.6 compared to tumors that were irradiated only [20]. In the present study, the potentiation of radiation response by ECT with BLM was 1.9 fold compared to tumors that were irradiated only and those that were concomitantly treated with BLM, since BLM only had no radiosensitising effect at this low drug dose. As discussed in our previous studies on ECT with cisplatin and irradiation, several mechanisms could be responsible for potentiation of radiation response of ECT with BLM [20,21].

First, electroporation induces transient permeability of cell membrane and therefore enables increased drug accumulation in the cells. For BLM as well as for cisplatin it was shown that electroporation of cells or tumors causes increased drug accumulation in tumor cells *in vitro *as well as in tumors [10,11,32]. Therefore, increased BLM accumulation in the tumor cells may consequently result in better radiosensitisation of cells and tumors.

Second, application of electric pulses to the tissues, either normal tissues or tumors results in temporary, but reversible reduction of tissue perfusion [16-19,23,33]. The effect was more pronounced in tumors compared to normal tissues, where it was shown that electroporation induced profound reduction of tumor blood flow and reduced tumor oxygenation [17-20,23]. In order to verify whether this kind of electric pulses induce hypoxia also in LPB tumor model, used in this study, tissue oxygenation was measured. It was shown that electroporation of tumors induced instant, but reversible tumor hypoxia, which was not restored at the time of tumor irradiation. Since the induced tumor hypoxia was radiobiologically relevant [34] it could have an effect on tumor response to irradiation. However, the curability of tumors treated with the electroporation combined with tumor irradiation was the same as the curability of tumors that were irradiated only. In contrast, our results *in vitro *demonstrated that electroporation of cells enhanced radiation response. Therefore some other mechanisms may affect the radiation response of tumors. It is already known that reactive oxygen species are formed after electroporation of the cells *in vitro *[35,36]. Reactive oxygen species are known to contribute to radiation damage to the cells. Indeed, we have observed in this and previous studies that electroporation of cells *in vitro *in normoxic conditions predisposed them to radiation damage [20,21]. In *vivo *on solid tumors, the radiation response was dependent on tumor type. Electroporation enhanced radiation response in EAT carcinoma, but not in LPB sarcoma [20,21]. Therefore, in this study, the observed radiation response of tumors that were exposed to electric pulses could be ascribed to, both radiobiologically relevant tumor hypoxia and induction of reactive oxygen species, the effects that counteract each other.

Consequently, in the case of ECT combined with irradiation, the electroporation-induced generation of reactive oxygen species in the tumor cells may additionally contribute to DNA damage induced by BLM and irradiation. However, since the data on reactive oxygen species were obtained on cells *in vitro *further studies *in vivo *are needed to demonstrate the presence and role of reactive oxygen species in tumors after electroporation. This question could be addressed also by prolonging the interval between the BLM or ECT and tumor irradiation, so as to ensure restitution of the tumor oxygenation before tumor irradiation is performed. Furthermore, this problem might be evaluated also by using anti-oxidant drugs in combination with electroporation and irradiation.

In conclusion, results of our study show that ECT with BLM greatly increased radiation response of LPB tumors. ECT is already used in treatment of patients with cutaneous and subcutaneous tumor nodules [13-15]. Recently, clinically certified generators of electric pulses came on the market. This will enable broader application of ECT in the clinics, also in combination with radiotherapy.

## Competing interests

The author(s) declare that they have no competing interests.

## Authors' contributions

SK participated in the design of the study, performed the experiments, and drafted the manuscript. MC participated in design of the study and critically revised the manuscript. AG participated in design of the study and experiments. MS helped to draft the manuscript and participated in analysis and interpretation of the data. GS conceived the study, participated in design of the study and coordination, and critically revised the draft.

## Pre-publication history

The pre-publication history for this paper can be accessed here:



## Figures and Tables

**Table 1:** Surviving fraction of the cells *in vitro*, tumor doubling time and complete responses of the tumors treated with BLM, electroporation and ECT.

***In vitro***			***In vivo***		
**Group**	**n**	**Sf^a^**	**n**	**DT (days)^b^**	**CR (n)^c^**
**Control**	9	1	16	2.3 ± 0.1	0
**BLM**	9	0.83 ± 0.03	17	4.4 ± 0.5	0
**EP^d^**	9	0.81 ± 0.02	13	4.9 ± 0.8	0
**ECT**	9	0.69 ± 0.03	23	19.6 ± 2.2	2

^a ^Surviving fraction of LPB cells

^b ^Tumor doubling time of the tumors that regrew after the treatment (AM ± SEM)

^c ^Tumor cures were determined 100 days after the treatment

^d ^Electroporation

**Table 2:** Comparison between tumor curability dose (TCD_50_), confidence interval (95%) and EF for irradiation alone (IR) or combined with electroporation (EP-IR), BLM (BLM-IR), and ECT (ECT-IR) of LPB sarcoma tumors.

**Group**	**TCD_50_**	**Confidence interval (95-%)**	**EF**
IR	23.1	22.6–23.6	
EP-IR	22.1	21.7–22.7	1.0
BLM-IR	22.8	22.4–23.4	1.0
ECT-IR	12.4	11.9–13.0	1.9
